# Comparison of capillary and venous blood for malaria detection using two PCR-based assays in febrile patients in Sierra Leone

**DOI:** 10.1186/s12936-021-03644-y

**Published:** 2021-03-06

**Authors:** Tomasz A. Leski, Chris Rowe Taitt, Umaru Bangura, Joseph Lahai, Joseph M. Lamin, Victoria Baio, Mohamed S. Koroma, Abdulai G. Swaray, Kathryn H. Jacobsen, Olivia Jackson, Brian W. Jones, Cynthia L. Phillips, Rashid Ansumana, David A. Stenger

**Affiliations:** 1 Center for Bio/Molecular Science & Engineering, U.S. Naval Research Laboratory, Washington, DC USA; 2 Mercy Hospital Research Laboratory, Bo, Sierra Leone; 3 College of Science, George Mason University, Fairfax, Virginia USA; 4 Department of Global & Community Health, George Mason University, Fairfax, Virginia USA; 5 BioFire Defense, Salt Lake City, Utah USA; 6 Njala University, Bo, Sierra Leone

**Keywords:** Malaria, Capillary blood, Venous blood, Multiplex polymerase chain reaction, *Plasmodium*, *Plasmodium falciparum*, *Plasmodium vivax*, *Plasmodium ovale*, Sierra Leone

## Abstract

**Background:**

Rapid and sensitive diagnostics are critical tools for clinical case management and public health control efforts. Both capillary and venous blood are currently used for malaria detection and while diagnostic technologies may not be equally sensitive with both materials, the published data on this subject are scarce and not conclusive.

**Methods:**

Paired clinical samples of venous and capillary blood from 141 febrile individuals in Bo, Sierra Leone, were obtained between January and May 2019 and tested for the presence of *Plasmodium* parasites using two multiplexed PCR assays: the FilmArray-based Global Fever Panel (GFP) and the TaqMan-based Malaria Multiplex Sample Ready (MMSR) assay.

**Results:**

No significant differences in *Plasmodium* parasite detection between capillary and venous blood for both assays were observed. The GFP assay was more sensitive than MMSR for all markers that could be compared (*Plasmodium* spp. and *Plasmodium falciparum*) in both venous and capillary blood.

**Conclusions:**

No difference was found in malaria detection between venous and capillary blood using two different PCR-based detection assays. This data gives support for use of capillary blood, a material which can be obtained easier by less invasive methods, for PCR-based malaria diagnostics, independent of the platform.

## Background

Malaria remains one of the most deadly infectious diseases in the world, disproportionately affecting lower-income countries in Africa and South Asia. According to current World Health Organization (WHO) estimates, more than 200 million malaria cases and more than 400,000 deaths are expected to occur this year, with the highest burden falling on young children in sub-Saharan Africa [1].

Sensitive and accurate diagnosis of malaria is the key to effective clinical case management and is an essential component of public health prevention and control strategies. Although diagnostic technologies using saliva and urine are being developed [2, 3], most current technologies continue to use whole blood collected via venipuncture or finger stick, and these materials are used interchangeably [4]. However, the chemical and cytological composition between blood from capillary and venous compartments differs [5–8], and these differences may potentially affect the sensitivity of malaria diagnostics. Sequestration of *Plasmodium*-infected erythrocytes in small capillaries of certain organs (e.g., brain, lungs)—a documented feature of malaria pathology [9]—may lead to unequal distribution of malaria parasites between capillary and venous compartments. That, in turn, may lead to different diagnostic results from these sample types.

Several previous studies assessed parasite density and the sensitivity of malaria detection in paired capillary and venous blood samples from asymptomatic carriers and symptomatic malaria patients in sub-Saharan Africa [4, 10–14]. Some comparisons using standard microscopic techniques and PCR showed no difference between capillary and venous samples [10, 12, 13], but others showed slightly higher parasite numbers in capillary specimens [4, 11, 14]. Additionally, one PCR-based study documented a greater diversity of strains detected in venous blood samples [10]. Due to the lack of consensus regarding whether sensitivity is different for capillary and venous samples, this area of diagnostic research merits further investigation. This study compared the sensitivity of malaria detection in paired capillary and venous samples from symptomatic hospital patients in Bo, Sierra Leone, using two different PCR-based assays capable of detecting multiple *Plasmodium* species: the FilmArray-based Global Fever Panel (GFP) and the TaqMan-based Malaria Multiplex Sample Ready (MMSR) assay.

## Methods

### Study population and sample collection

The study population was recruited among persons seeking care at Mercy Hospital in Bo, Sierra Leone. All persons with clinically confirmed or self-reported fever with onset within the 10 days before the enrollment date were invited to participate in the study. Informed consent from patients (or, for children, consent from their parents) was obtained and documented prior to collection of clinical data and biological specimens. In total, 141 volunteers were enrolled between 28 January and 20 May 2019. Paired samples of venous and capillary blood were collected into EDTA-containing vacutainers or microtainers from each participant by venipuncture and finger prick, respectively. The study protocol was approved by the Institutional Review Boards at the US Naval Research Laboratory and George Mason University, and by the Sierra Leone Ministry of Health and Sanitation.

### PCR-based analysis

Two PCR-based methods were used in this study: the FilmArray Global Fever Panel (GFP, BioFire Defense, Salt Lake City, UT, USA) and the Malaria Multiplex Sample Ready assay (MMSR, BioGX, Birmingham, AL, USA). The same volume (200 µL) of blood specimen was used in both systems and from both specimen types. GFP is a fully automated, nested PCR assay capable of automated extraction of nucleic acids from a blood sample and rapid (one hour) detection of nineteen targets using the FilmArray platform, including *P. falciparum*, *Plasmodium vivax*/*Plasmodium ovale*, and *Plasmodium* spp. [15]. Briefly, within two hours of sample collection, 200 µL of capillary or venous blood was mixed with 1 mL of GFP sample buffer. The entire diluted sample (total volume of 1.2 mL) was loaded into GFP pouches, and analysed on the BioFire FilmArray 2.0 instrument according to the manufacturer’s instructions. Detection and identification of the target is made automatically based on the melting temperature of the obtained amplicon. The validity of the run is determined by the system based on the amplification results of controls included in the assay pouch. The GFP has three different assays for *Plasmodium*: one species-level assay that detects *P. falciparum*, one species-level assay that detects both *P. vivax* and/or *P. ovale*, and one genus-level assay (*Plasmodium* spp.) that detects all *Plasmodium* species known to cause malaria in humans. The limits of detection (LOD) for GFP are 180, 150, and 240 genomic copies per mL blood for *P. falciparum*, *P. vivax*, and *P. ovale*, respectively (BioFire, unpublished data). The FilmArray system software calculates semi-quantitative crossing point values (the fractional PCR cycle number when fluorescence of the sample exceeds the background fluorescence threshold). The manufacturer of the instrument refers to these values as “Cp values.” The instrument’s software does not provide these values to the user but they can be accessed by the manufacturer and were used in this study to help interpret the data. Since Cp values and the more commonly used term “Ct values” are different names for the same value, the term “Ct values” is used for both amplification systems for the purposes of the discussion in this paper. Due to the differences in PCR reaction chemistries between GFP and MMSR assays (two-step nested PCR vs. one-step TaqMan PCR, respectively), the Ct values obtained using GFP assay are generally lower MMSR Ct values for the same analyte concentrations and cannot be directly compared between these systems.

The MMSR assay is a room temperature-stabilized TaqMan-based real-time PCR assay [16, 17], designed to detect *P. falciparum*, *P. vivax*, *Plasmodium* spp., and RNaseP (sample extraction control) in a single assay. While this assay was not specifically designed to detect *P. ovale* and other less common malaria species, these parasites may be identified in samples testing positive for the genus specific marker (*Plasmodium* spp.) and negative for both species specific markers (*P. falciparum* and *P. vivax*) [18]. Within two hours of collection, DNA was extracted from 200 µL capillary or venous blood using QIAamp DNA Mini Kit (Qiagen, Germantown, MD, USA); the final volume of extracted DNA was the same as the initial sample volume – 200 µL. Five microlitres of the extracted DNA was added to each MMSR tube, previously rehydrated with 5 µL water. Tubes were then subjected to a thermal cycling program as follows: initial incubation at 95°C for 2 minutes; 45 cycles of denaturation at 95°C for 10 seconds and annealing/elongation at 59°C for 1 minute. Fluorescence levels were measured at the end of each cycle. The samples were run using 8-tube strips and each run contained a negative (no template) and a positive (*P. falciparum* DNA) control. The run results were considered valid when all controls gave expected results. A sample was considered positive for a particular target in the MMSR assay if a sigmoidal amplification curve with Ct value < 40 was observed. The reported LODs for *P. falciparum* in the genus-specific assay (*Plasmodium* spp.) were 244–390 parasites per mL DNA solution, depending on whether the lyophilized or “wet” format was used [17]. The reported LOD in the *P. falciparum*-specific assay was similar in the lyophilized test (244 copies/mL), but was about 10-fold higher in the “wet” assay. The LOD for *P. vivax* was not reported for the lyophilized, “sample ready” format, but was previously determined for the “wet” assay as 127 parasites/mL [16].

Data from capillary or venous samples with negative RNaseP results (MMSR assay) or invalid FilmArray results were not included in the comparison. However, if the matched partner of the invalid sample showed valid results, data from the matched partner were included in statistical analyses of the population as a whole.

Sample populations returning valid results were compared using McNemar’s chi-square analysis of (self-)paired samples, corrected for continuity. Ct values for positive (matched) samples were compared using paired t-tests and for unmatched populations using unpaired t-tests, assuming unequal variances.

## Results

The median age of the 141 participants that provided blood samples was 27 years (range: 5 to 78 years), and 60 % were female. Depending on the assay and material tested, at least one malaria marker was detected in 36–61 % of the tested samples (Table 1). These results were in line with previous reports of malaria positivity among febrile individuals in this location as detected by PCR-based methods [18, 19].

**Table 1:** Malaria marker prevalence as detected by GFP and MMSR assays for paired venous/capillary samples

	Number (%) of positive samples	
	GFP (n = 66)	MMSR (n = 110)
Capillary		
* Plasmodium* spp.	37 (56 %)	29 (26 %)
* P. falciparum*	32 (48 %)	37 (34 %)
* P. vivax/P. ovale*^*a*^	4 (6 %)	0
Any malaria marker	38 (58 %)	40 (36 %)
Venous		
* Plasmodium* spp.	40 (61 %)	35 (32 %)
* P. falciparum*	30 (45 %)	37 (34 %)
* P. vivax/P. ovale*^*a*^	4 (6 %)	0
Any malaria marker	40 (61 %)	40 (36 %)

^a^GFP assay detects both *P. vivax* and *P. ovale* while MMSR assay detects only *P. vivax*

The study was designed to return four test results for each participant (two platforms, both venous and capillary samples tested on each). However approximately 63 % of the participants had one or more missing data points primarily due to insufficient sample volumes to perform both tests on both platforms or invalid runs (27 samples). The most common causes of the invalid runs were failures of the extraction control reactions for MMSR (3 samples) and hardware issues with one of the two FilmArray instruments used in case of GFP assay (18 samples). Consequently, valid results were returned for 110 paired capillary/venous blood samples using MMSR and for 66 paired samples using GFP. For cross-platform comparisons, venous samples from 136 individuals and capillary samples from 54 individuals were successfully tested on both platforms. The demographic and clinical characteristics of included and excluded volunteers were similar.

No significant age-specific differences were observed for any marker on either platform (Additional file 1: Table S1). Although no gender-specific differences were noted on the GFP platform, a significantly higher proportion of male participants were positive for *Plasmodium* spp. in the MMSR assay (p = 0.011); this result may simply represent an artifact of the large relative difference between male and female sample sets within a small total population (n = 136).

### Comparison of detection platforms

A total of 54 matched capillary and 136 venous blood samples returned valid results for both GFP and MMSR. The results for both detection platforms were compared in Table 2 for capillary and Table 3 for venous samples. GFP assays detected a significantly higher proportion of positive samples across all detected markers for both capillary and venous blood. The difference was particularly evident with the genus-level marker (*Plasmodium* spp.). The GFP platform detected *Plasmodium* spp. in over half of the samples (both sample types), whereas the same marker was detected in 22 % fewer samples analysed by MMSR. Fewer samples were positive for *P. falciparum* marker than *Plasmodium* spp. marker on the GFP, but cross-platform differences were still significant (*P. falciparum* was detected in 23/54 capillary samples by GFP compared to only 15/54 by MMSR, and 58/136 venous samples by GFP compared to only 47/136 by MMSR). In the case of MMSR assays, a smaller number of samples tested positive for *Plasmodium* spp. marker than *P. falciparum* marker for both sample types; this likely reflects previously reported differences in LOD values for these markers [16, 17]. Power analyses indicated that the sample sizes were sufficient to determine platform differences (80 % power, α = 0.05) in detection of *Plasmodium* spp. using any marker; however, sample sizes 1.3- to 1.6-times greater would be required for platform differences in detection of *P. falciparum* and any malaria marker with the same power and significance.

**Table 2:** Comparison of malaria detection by GFP and MMSR assays - capillary blood (n = 54); GFP is more sensitive than MMSR

Marker					p-value for McNemar’s χ^2^ test
*Plasmodium* spp.		MMSR			< 0.001 (χ^2^ = 13.474)
GFP		Positive	Negative	Total	
	Positive	10	18	28	
	Negative	1	25	26	
	Total	11	43	54	
*P. falciparum*		MMSR			0.043 (χ^2^ = 4.083)
GFP		Positive	Negative	Total	
	Positive	13	10	23	
	Negative	2	29	31	
	Total	15	39	54	
Any *Plasmodium* marker		MMSR			0.006 (χ^2^ = 7.563)
GFP		Positive	Negative	Total	
	Positive	14	14	28	
	Negative	2	24	26	
	Total	16	38	54	

**Table 3:** Comparison of malaria detection by GFP and MMSR assays—venous blood (n = 136); GFP is more sensitive than MMSR

Marker					*p*-value for McNemar’s χ^2^ test
*Plasmodium* spp.		MMSR			< 0.001 (χ^2^ = 25.037)
GFP		Positive	Negative	Total	
	Positive	44	27	71	
	Negative	0	65	65	
	Total	44	92	136	
*P. falciparum*		MMSR			0.022 (χ^2^ = 5.263)
GFP		Positive	Negative	Total	
	Positive	43	15	58	
	Negative	4	74	78	
	Total	47	89	136	
Any malaria marker		MMSR			< 0.001 (χ^2^ = 17.391)
GFP		Positive	Negative	Total	
	Positive	49	22	71	
	Negative	1	64	65	
	Total	50	86	136	

The GFP assay detected *P. vivax*/*P. ovale* in six out of 136 matched venous samples (4 %) and in four out of 54 matched capillary samples (7 %). The four capillary samples positive for *P. vivax*/*P. ovale* were simultaneously positive for this marker in venous samples. However, none of these samples were deemed positive for *P. vivax* in the MMSR assay, suggesting that the *P. ovale* may have been responsible for the *P. vivax*/*P. ovale*-positives in the GFP assay.

*P. ovale* (and *Plasmodium malariae*) may be detected, in the MMSR assay, when a sample is *Plasmodium* spp.-positive and negative for the species-specific markers [18]; only one of the samples positive by GFP for *P. vivax*/*P. ovale* demonstrated this behavior in the MMSR assay. Presumptively, samples harbouring *P. malariae* and other less-common species can be identified similarly by the MMSR assay (negative for *P. vivax*/*P. falciparum* and positive for *Plasmodium* spp.). This does not have to be true, however, for GFP because the *Plasmodium* spp. assay is slightly more sensitive than the GFP species-level assays (BioFire, unpublished data).

An additional nine venous and four capillary samples were identified that were *Plasmodium* spp.-positive but negative in the species-specific assays in GFP assays; all but two were negative for all markers in the corresponding MMSR assays. Interestingly, only three had identical results in both venous and capillary samples. All eleven of these samples that were only positive for *Plasmodium* spp. had late Ct values, suggesting analyte levels near LOD where it is expected that the *Plasmodium* spp. assay will slightly outperform the species-level assays on the GFP.

### Comparison of venous and capillary samples

**Fig. 1:**
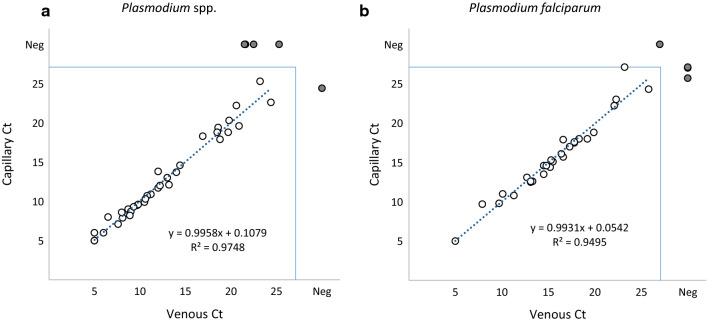
Comparison of Ct values obtained with venous (abscissa) and capillary (ordinate) samples positive for malaria markers after analysis using GFP. Panels A and B show Ct values for samples positive for *Plasmodium* spp. (**a**) and *P. falciparum* (**b**). Gray circles at the edges of each graph represent samples that were positive in venous but not matched capillary samples (above the main plot) or in capillary but not matched venous samples (to the right of each plot)

Results from matched sets of capillary and venous samples using GFP (Table 4) showed no significant differences in the proportion of samples testing positive for any of the malaria markers between venous and capillary samples. Fewer than 10 % of the 66 matched samples on GFP gave discordant results where a marker was positive in one sample type and negative in the other. Comparisons of Ct values between venous and capillary samples (Fig. 1) show a high degree of correlation when tested using GFP. There was no significant difference in Ct values between matched venous and capillary samples for any marker (p > 0.13).

**Table 4:** Comparison of malaria detection in capillary and venous blood—GFP assay (n = 66)

Marker					*p*-value for McNemar’s χ^2^ test
*Plasmodium* spp.		Capillary			0.371 (χ^2^ = 0.800)
Venous		Positive	Negative	Total	
	Positive	36	4	40	
	Negative	1	25	26	
	Total	37	29	66	
*P. falciparum*		Capillary			0.617 (χ^2^ = 0.250)
Venous		Positive	Negative	Total	
	Positive	29	1	30	
	Negative	3	33	36	
	Total	32	34	66	
*P. vivax/P. ovale*		Capillary			
Venous		Positive	Negative	Total	
	Positive	4	0	4	
	Negative	0	62	62	
	Total	4	62	66	
Any malaria marker		Capillary			0.617 (χ^2^ = 0.250)
Venous		Positive	Negative	Total	
	Positive	37	3	40	
	Negative	1	25	26	
	Total	38	28	66	

**Fig. 2:**
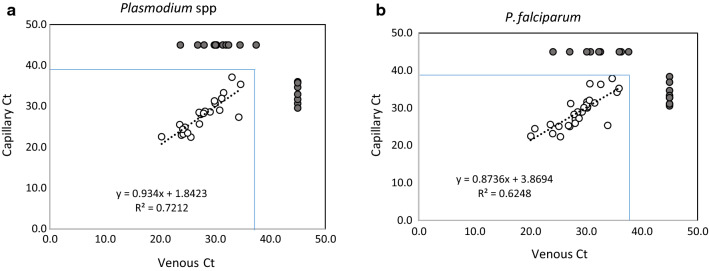
Comparison of Ct values obtained with venous (abscissa) and capillary (ordinate) samples positive for malaria markers after analysis using MMSR. Panels A and B show Ct values for samples positive for *Plasmodium* spp. (**a**) and *P. falciparum* (**b**). Gray circles at the edges of each graph represent samples that were positive in venous but not matched capillary samples (above the main plot) or in capillary but not matched venous samples (to the right of each plot)

Similarly, no significant differences between positive results were observed for any marker when using MMSR (p > 0.24, n = 110; Table 5). There was lower concordance between the two sample types in MMSR assays than in the GFP assays – 82 % for genus specific marker (*Plasmodium* spp.), 84 % for *P. falciparum* and 84 % for any malaria marker *versus* > 92 % for GFP. A lower correlation was observed when comparing Ct values for matched samples in MMSR assays (Fig. 2), although no significant differences in Ct values were observed, independent of the assay (p > 0.6). However, when comparing concordant populations (both sample types positive) and discordant populations (one sample type positive, one sample type negative), Ct values from the discordant populations were indeed higher (p < 0.015, see Fig. 1), supporting the hypothesis that samples with discordant results had lower target concentrations.

**Table 5:** Comparison of malaria detection in capillary and venous blood – MMSR assay (n = 110)

Marker					*p*-value for McNemar’s χ^2^ test
*Plasmodium* spp.		Capillary			0.264 (χ^2^ = 1.250)
Venous		Positive	Negative	Total	
	Positive	22	13	35	
	Negative	7	68	75	
	Total	29	81	110	
*P. falciparum*		Capillary			0.814 (χ^2^ = 0.0556)
Venous		Positive	Negative	Total	
	Positive	28	9	37	
	Negative	9	64	73	
	Total	37	73	110	
*P. vivax*		Capillary			
Venous		Positive	Negative	Total	
	Positive	0	0	0	
	Negative	0	110	110	
	Total	0	110	110	
Any malaria marker		Capillary			0.814 (χ^2^ = 0.0556)
Venous		Positive	Negative	Total	
	Positive	31	9	40	
	Negative	9	61	70	
	Total	40	70	110	

Percentages of discordant samples ranged from 1.5 to 6.1 % in the GFP assays and 6.4–11.8 % in the MMSR assays. Given similar prevalence of venous-positive/capillary-negative and venous-negative/capillary-positive pairs, power analyses indicated that we would need to test at least four times as many samples to show differences between the two sample types with α = 0.05 and 80 % power for all three GFP assays and MMSR-based *Plasmodium* spp. detection; identical rates of detection from both sample types in MMSR-based detection of *P. falciparum* and any malaria marker indicate that a much larger sample size (n > > 1000) would be needed to detect differences between sample types.

## Discussion

The goal of this study was to compare the sensitivity of DNA-based malaria diagnostic assays using capillary and venous blood. In contrast to most of the previous studies comparing these two sample types [4, 10–13], the sensitivity of two PCR assays capable of detecting multiple species of *Plasmodium* was assessed instead of determining the parasite density by microscopy. While PCR and other nucleic acid based assays are not currently used for routine malaria diagnostics in most countries, these techniques are increasingly used for epidemiological studies of malaria and there is a growing recognition of their important role in detecting subclinical malaria infections especially when coupled with strategic malaria elimination and eradication efforts. The analysis described here documented no significant differences in malaria detection between capillary and venous blood using two different PCR based assays.

These results are in line with several previous studies that also found no differences in parasite density or malaria detection between capillary and venous samples [10, 12, 13]. Comparisons of Ct values obtained using GFP and MMSR further support the hypothesis that there are no significant differences in average parasite densities between these two sample types.

The higher malaria detection rate observed for GFP compared to MMSR for all detected markers reflects fundamental and comprehensive differences between the two systems: different sample processing (nucleic acid extraction), different PCR chemistry, nested PCR (GFP) versus single-step PCR (MMSR), and different gene targets. Although the previously determined LODs for the two platforms are within the same range (~ 150–400 parasites or target copies per millilitre), a five-fold larger MMSR sample volume was used in the current study than in the MMSR LOD study (5 µL versus 1 µL [17]); it is possible that the increased sample volume, combined with a different method for DNA extraction, interfered with the MMSR assays performed here. The differences in sensitivity can be also a consequence of using different diagnostic targets; the effect is especially pronounced in case of the genus-specific targets (*Plasmodium* spp.). The samples with discordant results between sample types had significantly higher Ct values on both platforms; these results suggest that discordant results may arise when testing samples with parasite concentrations close to the LOD.

It was not possible to unambiguously assess the sensitivity differences in detecting *P. vivax* (MMSR) and *P. vivax*/*P. ovale* (GFP) due to the different specificities of the two platforms and the low prevalence of *P. vivax* within West Africa [1, 20, 21]. Importantly, two of the samples that were only positive for *Plasmodium* spp. by GFP in venous blood had relatively early Ct values that, if the infection were *P. falciparum, P. ovale*, or *P. vivax*, should also have been detected by one of the species-level assays; it is therefore likely that these samples contained *P. malariae*. The presence of small but significant numbers of samples positive for *P. vivax*/*P. ovale* in the GFP assay or *Plasmodium* spp. only in both assays could be due to co-circulation of *P. ovale* and *P. malariae* in the tested population as previously reported [18].

Three studies have previously reported higher parasite densities or improved malaria detection sensitivities in capillary blood [4, 11, 12]. The hypothesis that capillary blood may contain higher concentrations of malaria parasites is based on the well-documented feature of malaria pathology—increased adherence of *Plasmodium*-infected erythrocytes to vascular endothelial cells—which leads to their sequestration in certain organs [9]. However, while it is known that malaria-infected erythrocytes can sequester in specific organs (e.g., brain, lungs), it is not clear if significant sequestration occurs in capillaries used for diagnostic sample collection. The results obtained in this study do not provide support to the hypothesis that *Plasmodium* markers are present in higher concentrations in capillary blood, but rather, adds to the body of literature showing no documented difference between sample types.

A notable difference between the current and previous studies is the population age distribution. Whereas most other studies emphasized testing of children or young teens [4, 10, 11, 13, 14], our study population was significantly older, with a median of 27 years and 25 % of the tested individuals between 21 and 25 years of age. It is possible that age-related physiological differences may affect the parasite’s life cycle, its clinical presentation, and potentially even its prevalence within various compartments [22–24]. An age-controlled study with a greater number of participants and longer collection period would be needed to explore these variables.

## Conclusions

In summary, no difference in *Plasmodium* parasite detection sensitivity between capillary and venous blood was found. While accepting the limitations of the current study (small sampled population with a different age distribution from previous studies), the results presented here provide additional supporting evidence that PCR-based methods can produce equally satisfactory results using capillary and venous blood, in spite of the higher diagnostic sensitivity of capillary blood suggested by some recent studies. The implication of this finding is that capillary blood—typically less cumbersome and invasive to obtain — can be used without a loss of the assay sensitivity. Additional, larger-scale studies are needed to support these conclusions.

## Supplementary Information

**Additional file 1: Table S1**. Samples positive for various malaria markers by age and gender

## Data Availability

The datasets used and/or analyzed during the current study are available from the corresponding author on reasonable request.

## Abbreviations

Cp: Crossing point.
Ct: Crossing threshold.
GFP: Global Fever Panel.
MMSR: Multiplex Malaria Sample Ready assay.
LOD: Limit of detection.
PCR: Polymerase chain reaction.
WHO: World Health Organization.
