# Supplementary material for: Comparison of capillary and venous blood for malaria detection using two PCR-based assays in febrile patients in Sierra Leone
Source: Malar J. 2021 Mar 6;20:133. doi: 10.1186/s12936-021-03644-y (PMC7936501; doi:10.1186/s12936-021-03644-y)
Supplement: Supplementary file 1 — Additional file 1: Table S1. Samples positive for various malaria markers by age and gender [file 12936_2021_3644_MOESM1_ESM.docx]

Supplementary table. Samples positive for various malaria markers by age and gender.

| MMSR | | | | | | | | | | | | | | |
| --- | --- | --- | --- | --- | --- | --- | --- | --- | --- | --- | --- | --- | --- | --- |
| age (yrs) | venous | | | | | | | capillary | | | | | | |
|  | total valid | # positive for | | | | | | total valid | # positive for | | | | | |
|  |  | *P. falciparum* | p | *Plasmodium* spp. | p | *P. vivax* | p |  | *P. falciparum* | p | *Plasmodium* spp. | p | *P. vivax* | p |
| 5-15 | 19 | 6 | 0.260* | 6 | 0.260 | 0 | -- | 13 | 5 | 0.085 | 5 | 0.207 | 0 | -- |
| 16-30 | 64 | 25 |  | 24 |  | 0 |  | 53 | 23 |  | 17 |  | 0 |  |
| 31-45 | 27 | 5 |  | 4 |  | 0 |  | 23 | 5 |  | 4 |  | 0 |  |
| 46+ | 28 | 11 |  | 11 |  | 0 |  | 23 | 4 |  | 3 |  | 0 |  |
| F | 84 | 24 | 0.150 | 20 | 0.011 | 0 | -- | 63 | 18 | 0.310 | 15 | 0.660 | 0 | -- |
| M | 54 | 23 |  | 24 |  | 0 |  | 49 | 19 |  | 14 |  | 0 |  |
| GFP | | | | | | | | | | | | | | |
| age (yrs) | venous | | | | | | | capillary | | | | | | |
|  | total valid | # positive for | | | | | | total valid | # positive for | | | | | |
|  |  | *P. falciparum* | p | *Plasmodium* spp. | p | *P. vivax/P. ovale* | p |  | *P. falciparum* | p | *Plasmodium* spp. | p | *P. vivax/P. ovale* | p |
| 5-15 | 19 | 7 | 0.230 | 10 | 0.170 | 1 | 0.580 | 11 | 5 | 0.780 | 7 | 0.750 | 1 | 0.75 |
| 16-30 | 64 | 33 |  | 38 |  | 4 |  | 30 | 15 |  | 17 |  | 2 |  |
| 31-45 | 29 | 9 |  | 10 |  | 0 |  | 14 | 5 |  | 6 |  | 0 |  |
| 46+ | 27 | 10 |  | 13 |  | 1 |  | 13 | 7 |  | 7 |  | 1 |  |
| F | 84 | 34 | 0.730 | 41 | 0.600 | 2 | 0.210 | 42 | 19 | 0.800 | 22 | 0.800 | 1 | 0.15 |
| M | 55 | 24 |  | 30 |  | 4 |  | 26 | 13 |  | 15 |  | 3 |  |

* p > 0.05 is not significant.
